# Quaternion Wavelet Transform and a Feedforward Neural Network-Aided Intelligent Distributed Optical Fiber Sensing System

**DOI:** 10.3390/s23073637

**Published:** 2023-03-31

**Authors:** Lei Fan, Yongjun Wang, Hongxin Zhang, Chao Li, Xingyuan Huang, Qi Zhang, Xiangjun Xin

**Affiliations:** 1 State Key Laboratory of Information Photonics and Optical Communications, School of Electronic Engineering, Beijing University of Posts and Telecommunications (BUPT), Beijing 100876, China; fanlei@bupt.edu.cn (L.F.);; 2 Beijing Key Laboratory of Space-Round Interconnection and Convergence, Beijing 100876, China

**Keywords:** distributed optical fiber sensor network, Brillouin optical time-domain analysis, quaternion wavelet transform, depth feedforward neural network, Brillouin frequency shift retrieval

## Abstract

In this paper, aiming at a large infrastructure structural health monitoring network, a quaternion wavelet transform (QWT) image denoising algorithm is proposed to process original data, and a depth feedforward neural network (FNN) is introduced to extract physical information from the denoised data. A Brillouin optical time domain analysis (BOTDA)-distributed sensor system is established, and a QWT denoising algorithm and a temperature extraction scheme using FNN are demonstrated. The results indicate that when the frequency interval is less than 4 MHz, the temperature error is kept within ±0.11 °C, but is ±0.15 °C at 6 MHz. It takes less than 17 s to extract the temperature distribution from the FNN. Moreover, input vectors for the Brillouin gain spectrum with a frequency interval of no more than 6 MHZ are unified into 200 input elements by linear interpolation. We hope that with the progress in technology and algorithm optimization, the FNN information extraction and QWT denoising technology will play an important role in distributed optical fiber sensor networks for real-time monitoring of large-scale infrastructure.

## 1. Introduction

Large-scale infrastructures, such as railways, dams, bridges, oil pipelines, and power equipment, are an important pillar of economic development. In order to ensure the safe operation of large-scale infrastructure, it is necessary to carry out life-cycle structural monitoring and carry out appropriate maintenance schemes [1]. The planning and construction of many major construction projects also rely on the historical records and analysis of many large-scale infrastructures’ structure monitoring [2]. For large facilities such as bridges and buildings, temperature parameters are key data related to the safety, applicability, and stability of buildings. Real-time temperature measurement of facilities is a key technology to ensure that facilities are put to use. In recent years, the development of the Internet of things, the industrial Internet, big data, cloud computing and other technologies provides real-time or quasi real-time monitoring opportunities for large-scale infrastructure structure monitoring. Data acquisition for large-scale infrastructure structural health monitoring usually depends on a distributed sensing system. For a distributed system covering tens of kilometers, the amount of raw data obtained by measurement at any one time is usually very large [3]. Although the network capacity has been greatly developed in recent years, it is not a wise choice to upload massive amounts of original data. In order to reduce the network burden, a feasible scheme is to extract useful information from the original data and upload it to the data center [4]. An effective approach is needed in order to know the time-varying distribution of temperature-induced strain fields based on discrete sensing points [5].

However, it is a very cumbersome work to extract information from massive amounts of raw data from a distributed system using traditional data-processing methods, which usually introduce a lack of real-time performance or a decline in detection accuracy. The former may lead to the failure of the early warning function, while the latter may give rise to a wrong alarm or wrong decision. For example, as a typical representative of a distributed sensor system, a Brillouin optical time domain analysis (BOTDA) sensor system is expected to be widely used in the health monitoring of large facilities due to its low price, immunity to electromagnetic interference and ability to supply remote power [6,7].

**Figure 1:**
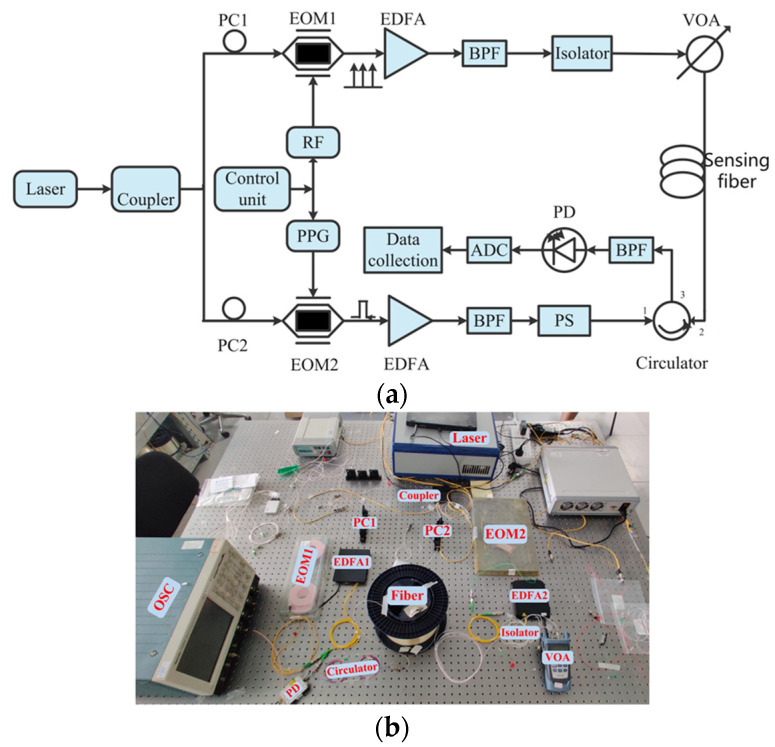
BOTDA distributed sensing system, (**a**) schematic diagram and (**b**) physical diagram.

The BOTDA system model we built is shown in Figure 1. The wavelength of continuous light emitted by the laser is 1550 nm, and the bandwidth is 10 Hz. The continuous light passing through the optical coupler is divided into two parts, which pass through the electro-optic modulators EOM1 and EOM2, respectively. For the upper branch, the microwave synthesizer dynamically changes the RF in the range of 10.76 GHz to 10.96 GHz, in steps of 1 MHz, to control EOM1. For the lower branch, the pattern generator is programmed to generate a high extinction ratio optical pulse sequence, with a time width of 30 ns and a repetition frequency of 4 kHz to control the EOM2. The continuous probe light and pump pulse light from the upper and lower branches are amplified by the erbium-doped fiber amplifier, and then passed through the bandpass filter. The power of the continuous detection light of the upper branch at the input end of the sensing fiber is adjusted to 8 dBm through the isolator and variable optical attenuator. The pump pulse light of the lower branch passes through the polarization scrambler, and is input to the sensing fiber through the optical ring; its input direction is opposite to that of the detection continuous light. At the end of the sensing optical fiber, a section of 200 m long fiber is put into the incubator, and the rest of the fiber is kept at room temperature. The data are processed and collected at the receiving end.

For a 40 km-long BOTDA high-spatial resolution monitoring system, the amount of raw data measured at one time is more than 100 m bytes, while each data acquisition time is tens of milliseconds. Network congestion from uploading original data is inevitable. Up to now, some conventional methods such as searching peak point, cross-correlation method (XCM), and Lorentzian curve fitting (LCF), have been proposed to extract the Brillouin frequency shift (BFS) distribution from the collected original Brillouin gain spectrum (BGS), which can be obtained by sweeping the frequency of the probe light and detecting the intensity gains of the probe signal. The peak search method and XCM can be carried out in negligible time, but a poor measurement accuracy will be obtained. Although the accuracy of BFS can be guaranteed using the LCF method, LCF is very time-consuming. In order to extract the physical information distribution with high precision and high spatial resolution at the cost of as little time as possible, in this paper, we try to introduce an artificial neural network (ANN) into structural health monitoring of large-scale infrastructures, to extract physical information directly from original data instead of traditional methods.

However, with the collected original data such as the raw BGS often being drowned in severe noise, it is difficult to achieve accurate temperature/strain from the noisy BGS, even if the collected BGS are averaged hundreds of times. Therefore, before BGS data are input to ANN, a proper denoising algorithm must be carried out. In recent years, many conventional signal-processing techniques, such as wavelet denoising (WD) [8] and the lifting wavelet transform (LWT) [9], have been used for BGS denoising and can be implemented in few seconds, but the effect of denoising is not enough to support high-precision measurement. Recently, some image-processing techniques [10,11,12] and an ANN [12,13] have been introduced to denoising BGS images, and a remarkable SNR enhancement has been demonstrated. However, non-local means (NLM) and block-matching and 3D image-filtering denoising methods are time-consuming, and often bring about degradation of spatial resolution. The collected BGS has been averaged dozens or even hundreds of times to reduce random noise before being input into an ANN module, and the training process is a time-consuming and tedious task. In this paper, a quaternion wavelet transform (QWT) image denoising technique is introduced into BGS image denoising, before BGS data are input to a depth feedforward neural network (FNN) module. The QWT image denoising not only eliminates noise, but also preserves more useful image details [14].

High time efficiency and good performance can be achieved by comprehensive utilization of QWT denoising and FNN technologies [15]. This paper focuses on the pre-processing of original data and the extraction of physical information. The FNN information extraction scheme and the QWT image denoising algorithm proposed in this paper have universal significance in the structural health monitoring of large-scale infrastructure.

The main contributions of this paper are as follows:

First, we have established the BOTDA system to meet the core functions required for the background of temperature sensing in large infrastructure. Then, we focused on the QWT algorithm part, which is used to denoise incoming raw BGS data for further processing. Finally, we established a feedforward artificial neural network and transferred the denoised BGS data into training to obtain temperature information.

## 2. QWT Image Denoising

The original data of structural health monitoring come from a BOTDA experimental system, with a fiber under test (FUT) around 40 km long. At the end of the FUT, a section of about 200 m is put into an incubator, while the rest is kept at room temperature. The scanning frequency interval varies from 10.77 GHz to 10.96 GHz. The time-domain width of the pump pulse is 15 ns, and the repetition rate is 4 kHz. The probe light is detected by a 125 MHz high-transimpedance photodetector and collected by an oscilloscope (OSC). The collected results are averaged according to the required times and saved in the OSC’s memory. The BGS image processed by QWT and containing 200 × 100,000 pixels is mapped from raw BGS, using a 500 MHz sampling rate, 16 times average, and a frequency range of 200 MHz with a step of 1 MHz. In this paper, BGS pixels are processed in matrix form. For the relationship between the BGS data and BGS pixels, the 200 × 100,000 pixel BGS image is composed of 100,000 data lines, represented by BGS values arranged in sequence on the horizontal axis coordinate. In this three-dimensional image, the horizontal axis coordinate represents the position of the sensing optical fiber, and the perpendicular axis coordinate denotes the frequency difference between the pump and probe Δυ; meanwhile, the axis perpendicular to the horizontal plane and representing Brillouin gain is described as a function of the position coordinates.

As a new kind of multiresolution analysis tool for image processing, QWT is approximately shift-invariant. In order to use the QWT denoising technique to process a BGS image, the image decomposition based on QWT must be performed. The low-frequency sub-band (L) and high-frequency sub-band (L) are obtained by a one-dimensional discrete wavelet transform, according to the row of original BGS images, and these constitute the data block of the first wavelet decomposition, with the L sub-band at the left, and the H sub-band at the right. Then, one low-frequency sub-band (LL), and three horizontal (HL), vertical (LH) and diagonal (HH) sub-bands are formed by a second one-dimensional discrete wavelet transform, according to the column of the results of the first wavelet decomposition. The second wavelet decomposition of the BGS image is obtained with the LL sub-band at the top left of the data block, while the HL, LH and HH sub-bands are at its top right, bottom left and bottom right, respectively. The coefficient for the four sub-bands is expressed by the real function $f\left(x,\;y\right)$.

On this basis, the QWT adopts four real discrete wavelet transforms. The first real discrete wavelet *f(x,y)* corresponds to the real part of quaternion wavelet, the second and third real discrete wavelets are obtained by the partial Hilbert transformation along the horizontal and vertical direction of data block of the second wavelet decomposition, and the last one is obtained by a whole Hilbert transform. The transformed result can be expressed as

(1) $$f_{qwt}\left(x,\;y\right)=f\left(x,\;y\right)+iH_{Hx}\left[f\left(x,\;y\right)\right]+jH_{Hy}\left[f\left(x,\;y\right)\right]+kH_{Hxy}\left[f\left(x,\;y\right)\right]$$

The last three parts correspond to three imaginary parts of quaternion wavelet, respectively. The quaternion wavelet can be expressed by one amplitude and three phases as

(2) $$f_{qwt}\left(x,\;y\right)=\left|f_{qwt}\left(x,\;y\right)\right|e^{i\phi }e^{j\theta }e^{k\psi }\;$$

**Figure 2:**
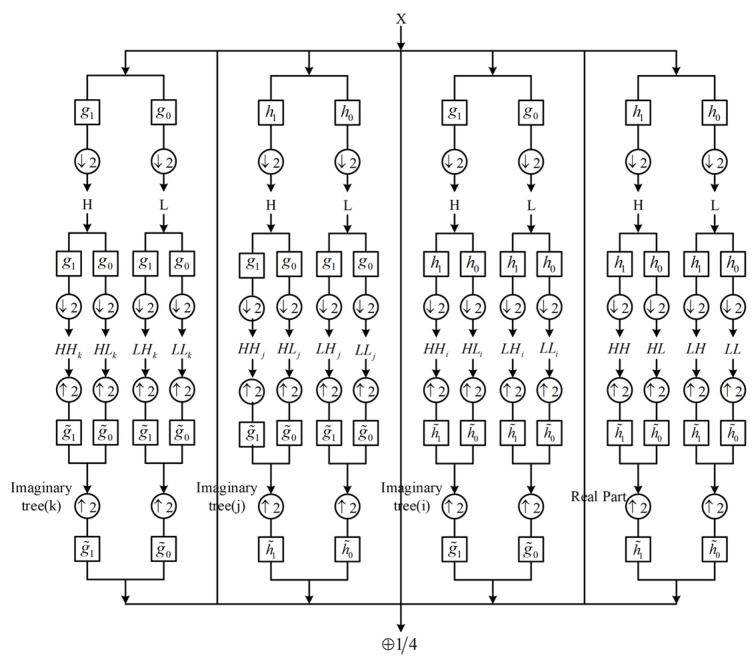
Flow chart of QWT algorithm.

**Figure 3:**
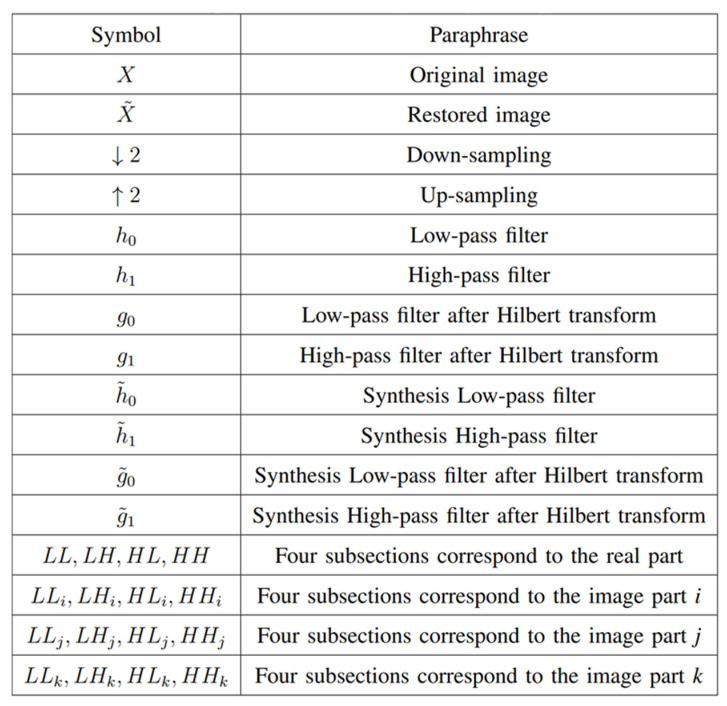
Symbols and meanings for QWT algorithm.

We took distance and frequency as horizontal and vertical coordinates to reduce the dimensions of the BGS 3D pixel data to 2D data. The BGS data after quaternion wavelet decomposition can be represented as one amplitude image and three phase images, as shown in Figure 2. Some relevant symbols and their meanings are shown in Figure 3.

The noise variance $\sigma_{n}$ according to the robust estimation can be calculated as

(3) $$\sigma_{n}=\frac{Median\left(\left|\omega_{i,j}\right|\right)}{0.6745\;}$$

and the amplitude variance can be calculated as

(4) $$\sigma_{w}^{2}=\frac{1}{N^{2}}\sum_{\;\left|\omega_{j}\right|\in W\left(k\right)}{\left(\left|\omega_{j}\right|\right)}^{2}$$

where $\omega_{i,j}$ is the best descendant coefficient, $\left|\omega_{i,j}\right|$ represents its amplitude value, $W\left(k\right)$ is the square adjacent window, $\mathrm{and}\;\omega_{j}$ and $N^{2}$ are the attribute values of the window. The variance $\sigma_{m}$ can be calculated as

(5) $$\sigma_{m}=\sqrt{max\left(\sigma_{w}^{2}-\sigma_{n}^{2},0\right)}\;$$

The amplitude value of a reasonable denoising coefficient can be calculated by the Bayesian shrinkage threshold as

(6) $$T=\frac{\sigma_{n}^{2}}{\sigma_{m}}$$

The coefficient matrix *m* can be calculated as

(7) $$m_{k,j}^{i}=sgn\left(w_{k,j}^{i}\right)\times \left(\left|w_{k,j}^{i}\right|-T_{\;k,j}^{i}\right),\;if\left|w_{k,j}^{i}\right|>T_{k,j}^{i}$$

where $w_{k,j}^{i}$ is the ith coefficient in the j direction of layer *k*, $T_{\;k,j}^{i}$ is the corresponding threshold of $w_{k,j}^{i}$, $\mathrm{and}\;m_{k,j}^{i}$ is the corresponding coefficient after the denoising of $w_{k,j}^{i}$.

After the amplitude image is de-noised through the above process, the inverse QWT operation is performed by using the estimated amplitude and the original phase, and the QWT coefficients are obtained by up-sampling twice to obtain the de-noised BGS image.

**Figure 4:**
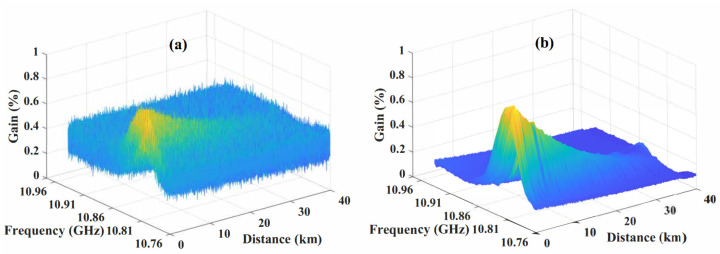
GS image from raw BGS data (**a**) and by QWT image denoising (**b**).

The three-dimentional raw BGS image and corresponding denoised image are shown in Figure 4.

**Figure 5:**
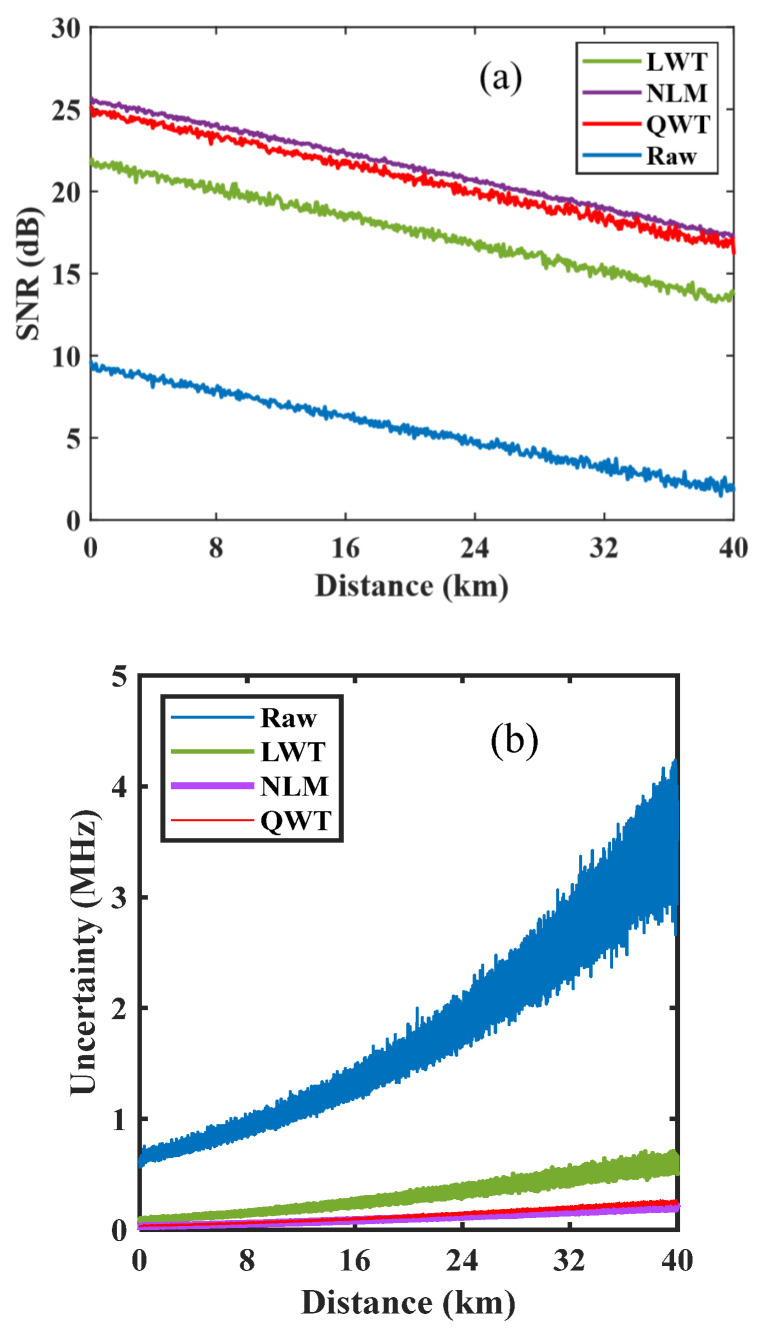
Denoising evaluation by calculated SNR (**a**) and frequency uncertainty of retrieved BFS (**b**).

The denoising effect of QWT can be assessed by calculating the SNR along the FUT. The SNR is obtained through the time–domain trace. The peak Brillouin gain frequency in the time–domain trace is as the signal amplitude, while noise is roughly estimated by the standard deviation of the non-gain area in the time–domain trace. The corresponding calculation results are shown in Figure 5a. At the end of the FUT, the SNR from raw data is 2.14 dB, and is 13.56, 17.24, and 16.86 dB from denoised BGS images by LWT, NLM, and QWT, respectively. In comparison with LWT, an obvious SNR enhancement of 3.1 dB has been achieved by using QWT. The SNR with QWT is only 0.38 dB lower than it is with NLM.

The fluctuation of the retrieved BFS is defined as frequency uncertainty, which can be estimated according to the method in [13]. The frequency uncertainty along the FUT is calculated and shown in Figure 5b. At the end of FUT, the frequency uncertainty is 4.1 MHz, obtained from raw BGS, and is 0.71, 0.19, and 0.21 MHz when using the LWT, NLM and QWT denoising methods, respectively. In the aspect of stability, the QWT has obvious advantages over LWT, and is similar to NLM.

**Figure 6:**
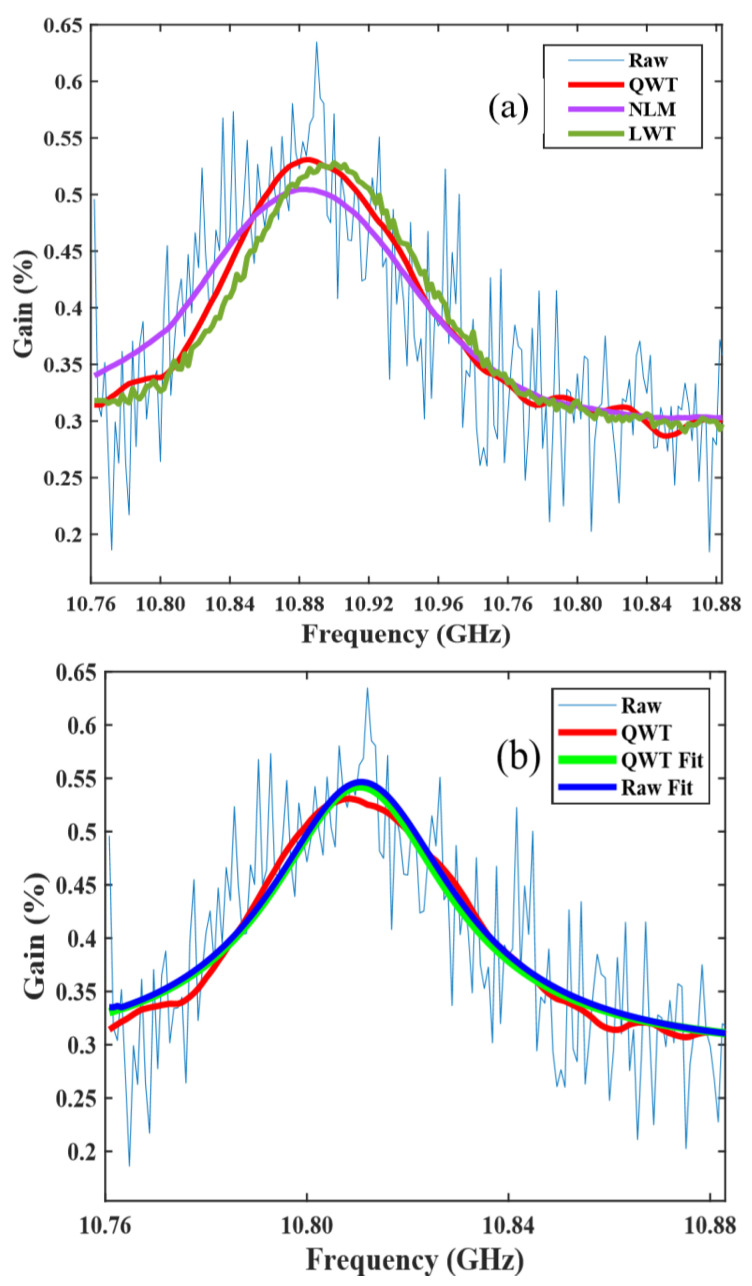
Different BGS curves (**a**) and BGS curves by LCF (**b**).

Subsequently, the BFS accuracy of the denoised BGS images is investigated. At a FUT of 15 km, the raw BGS curve and BGS curves using LWT, NLM and QWT are depicted and shown in Figure 6a. Two curves obtained using NLM and QWT exactly match the original BGS contour, while a slight shift between the original BGS curve and that processed by LWT is observed. Figure 6b shows two BGS curves, one obtained using LCF with an original curve, and another obtained using LCF with a denoised one by QWT. The BFS values from two BGS curves by LCF are 10.8230 GHz, corresponding to 23 °C, which is fully in accordance with the ambient temperature. However, it must be pointed out that when initial values of parameters required for the LCF iteration process are determined by BGS at the front end of the FUT, the fitted BGS curves at the room temperature zone match the original BGS contour, but in the heating zone, two curves may no longer match. The reason for this mismatch is that the parameters required by LCF cannot be selected dynamically according to different raw BGS curves. Especially at the end of FUT, the amplitude of BGS is very small, and almost submerged in noise. It is difficult to extract the maximum value and FWHM from such a noisy image. On the contrary, for the BGS denoised by QWT, it is easy to obtain an accurate maximum amplitude and FWHM. Almost all BGS curves obtained by LCF with a denoised image by QWT match their corresponding original shapes, when the fitting parameters are selected dynamically in accordance with the denoised BGS curves.

For extracting the temperature information from the denoised BGS image using a neural network, we can use the following formula to calculate the corresponding temperature:

(8) $$\upsilon_{B2}-\upsilon_{B1}=C_{T}\left(T_{2}-T_{1}\right),$$

where *υ_B_*_1_ and *υ_B_*_2_ represent the BFS retrieved from BGS curve 1 and BGS curve 2, which can be derived from the FNN, respectively. Moreover, *T*_1_ and *T*_2_ denote the temperature for BGS curve 1 and curve 2 at different points of the FUT, respectively. *C_T_* is the temperature coefficient, set as 0.975 MHz/°C in our experimental system.

**Figure 7:**
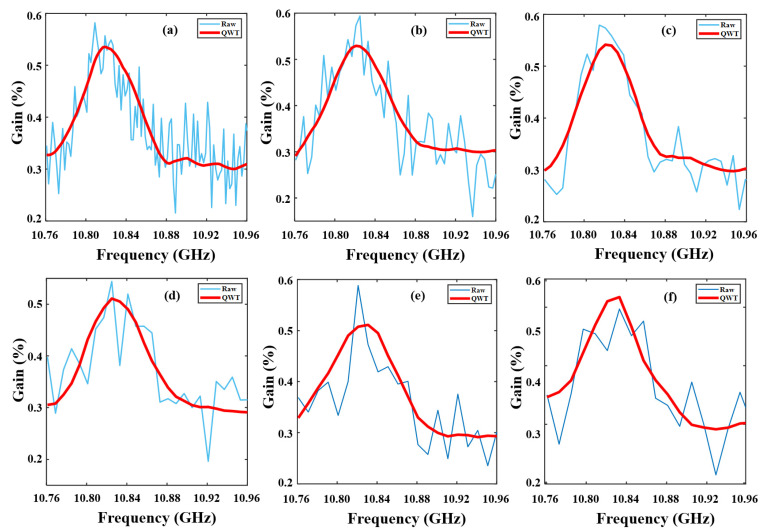
GS curves with different frequency intervals (**a**) 2M, (**b**) 4M, (**c**) 6M, (**d**) 8M (**e**) 10M and (**f**) 12M.

Then, we study the adaptability of the QWT to scanning intervals. BGS images were collected using 2, 4, 6, 8, 10, and 12 MHz scanning intervals. The sampling rate dropped from 500 MHz to 200 MHz. The raw BGS curves at 15 km of the FUT and the corresponding results denoised by QWT are shown in Figure 7. When the scanning interval is 2 MHz, using LCF, the BFS extracted from BGS and denoised by QWT is 10.8224 GHz; it is 10.8226 GHz and 10.8229 GHz for 4 MHz and 6 MHz, corresponding to temperatures of 22.39, 22.59 and 22.89 °C respectively, which are in accordance with room temperature (22.5 °C). The BFS deviation is about 0.38 MHz if an 8 MHz scanning interval is adopted. When the scanning interval exceeds 10 MHz, it is difficult to obtain a smooth curve from the finite sampling points using QWT. The spatial resolution will degrade with the sampling rate. A 200 MHz sampling rate corresponds to 1 m spatial resolution, which meets the detection requirement of many large infrastructures.

The effectiveness of the QWT denoising algorithm can be assessed by its running time in a computer. For a raw BGS image with 200 × 100,000 pixels, the running time of the LWT, NLM and QWT denoising algorithms in a PC equipped with an eight-core processor operating at 3.66 GHz is shown in Table 1.

The LWT algorithm has the highest operation efficiency, but it introduces slight frequency deviation. The NLM algorithm has advantages in SNR and frequency uncertainty, but it is time-consuming and brings spatial resolution degradation. Although the QWT algorithm takes 32 s to denoise the raw BGS image, it shows good performance in SNR measurement accuracy.

Moreover, importantly, when the scanning interval is changed to 4 MHz, there is no significant decrease in the measurement accuracy. Even if the scanning interval is increased to 6 MHz, the accuracy of the BFS can still reach ±0.15 MHz, meaning it can meet the detection requirement of many large infrastructures. It takes less than 2 s to process a BGS image with 33 × 40,000 pixels, which provides the possibility of quasi-real-time measurement.

## 3. Temperature Extraction from FNN

In the previous content, we only discussed the implementation of the QWT image denoising method and its corresponding denoising effect and running efficiency; the final BFS or required measured temperature/strain distribution has not been determined. The retrieval of BFS by peak-seeking can be executed in imperceptible time, but it is difficult to obtain accurate measurement results. The XCM also has higher efficiency, and when the appropriate Lorentz curve is selected, the retrieved BFS accuracy can be guaranteed. However, the Lorentz curve must be constructed dynamically according to the FWHM of the collected BGS image, otherwise the measurement accuracy will not be guaranteed. LCF is also a time-consuming task; it takes about 11 m to perform the LCF for the image mentioned above. In order to ensure measurement accuracy and time efficiency, the popular ANN may be an effective scheme to implement BFS’ retrieval or the extraction of temperature/strain from a BGS denoised by QWT.

The FNN has a very powerful fitting ability and can be used as a universal function. Through complex feature transformation, it can approximate any bounded closed set function with any accuracy. In the FNN, each layer of neurons can receive the signals from the previous layer of neurons and generate signals to output to the next layer. The first layer is called the input layer, the last layer is the output layer, and the other middle layer is the hidden layer. In the process of training, the number of neurons in each layer and the corresponding parameters are determined by back feedback, but after the network structure is determined, there is no feedback in the whole network, and the signal propagates from the input layer to the output layer in one direction during the processing of test sets. So, the FNN is suitable for extracting the temperature/strain distribution from the BGS at a very fast speed. The number of neurons, weight matrices and bias values for all layers can be determined in the training process. The gradient descent algorithm for backpropagation (BP) is used to minimize errors in the training process.

Theoretically, the ideal BGS curve conforms to the shape of the Lorentzian curve [16]. The training set is constructed according to the standard Lorentzian curve. The change in the BFS has a linear relationship with temperature [17]. Before the training set is created, the temperature coefficient must be determined by experiment. In our experimental system, the temperature coefficient of the FUT is determined as 0.975 MHz/°C. In the traditional training process, noise is added to the training data to enhance the applicability of the ANN, so the standard Lorentzian curves with different levels of noise are used as the training dataset [13,18]. In order to ensure a wide temperature detection range, the measured BGS needs to be scanned over a wide enough frequency range of 200 MHz, and the corresponding scanning frequency varies from 10.77 GHz to 11.96 GHz. According to the previous analysis, when the scanning frequency interval is less than 6 MHz, the accuracy of the retrieved BFS is ±0.15 MHz. So, the corresponding frequency intervals are determined as 1, 2, 3, 4, 5 and 6 MHz in the training process. When the experimental condition and the measurement range of high temperature-resistant fiber being considered, the temperature range for training is determined as 0 to 100 °C, and the temperature step is set to 0.1 °C. The shape of the BGS curve also depends on FWHM, so the BGS being trained for each temperature has a variable linewidth, which is designated from 30 to 70 MHz, with a step of 1 MHz. According to the calculated results, the SNR is about 3 dB at the end of the FUT for the raw BGS data, and about 26 dB at the start of the FUT for the BGS data using NLM. The SNRs range from 2 dB to 29 dB, and a step of 3 dB for the training BGS data is considered reasonable. Therefore, the total amount of required BGS curve data for the training process is 1001 × 41 × 10 × 6 = 2,462,460. Moreover, corresponding to the 1, 2, 3, 4, 5, and 6 MHz scanning intervals, the number of input elements is 200, 100, 67, 50, 40, and 33. So, six different FNN modules are required for different input vectors. For the scanning interval of 1 MHz, the input vector $X=\left[x_{1},x_{2},\ldots ,x_{200}\right]$ for the training process is a gain vector of 200 simulated scanning frequencies, and must be normalized. The temperature can be set as the output of FNN module. In the training process, multilayer layers of BP networks with 200 importing units and one exporting unit are set, a different number of hidden layers and nodes are selected to train, and then the relative errors are compared to confirm the neural network’s structure. After several attempts, the FNN layout of (200-80-20-1) is found to be enough for acceptable results. The same process continues until six modules with different input vectors are completed. This training process is a time-consuming and tedious task, and usually takes several days.

**Figure 8:**
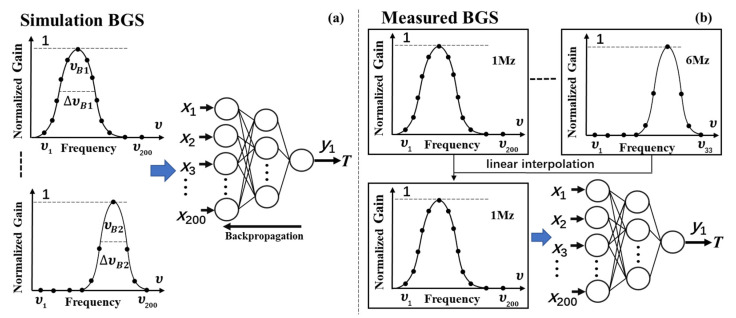
Training (**a**) and testing (**b**) schematic of the FNN.

However, the excellent denoising effect and good shape-matching of the QWT denoising technique provide a basis for simplifying the training process. In our scheme, as shown in Figure 8, contrary to conventional schemes with noisy Lorentzian curves as the training dataset, ideal Lorentzian curves with a scanning interval of 1 MHz are used as the training dataset. The total amount of required BGS curve data for the training process is reduced to 1001 × 41 = 41,041. The final number of modules is decreased from six to one, and a corresponding FNN layout of (200-50-10-1) is determined. The training process is carried out in only several tens of minutes. Instead of raw BGS data as the input to the FNN module, in our scheme, the BGS data processed by the QWT denoising algorithm are supplied to the FNN module. To eliminate the difference between input vectors and the import interface of the FNN module, the input vectors *X* must be unified into 200 importing elements through linear interpolation, which can be carried out after the QWT denoising process is applied to the raw BGS image. According to the previous analysis, when the scanning interval is less than 6 MHz, linear interpolation cannot induce evident degradation of the BFS accuracy. The input BGS data are also required to be normalized to adapt the training dataset. The FNN model can learn the relationship between the BGS and the temperature after the training process, and then the trained FNN module can be used to extract the values of temperature directly from the denoised BGS images processed by the QWT denoising algorithm and linear interpolation.

**Figure 9:**
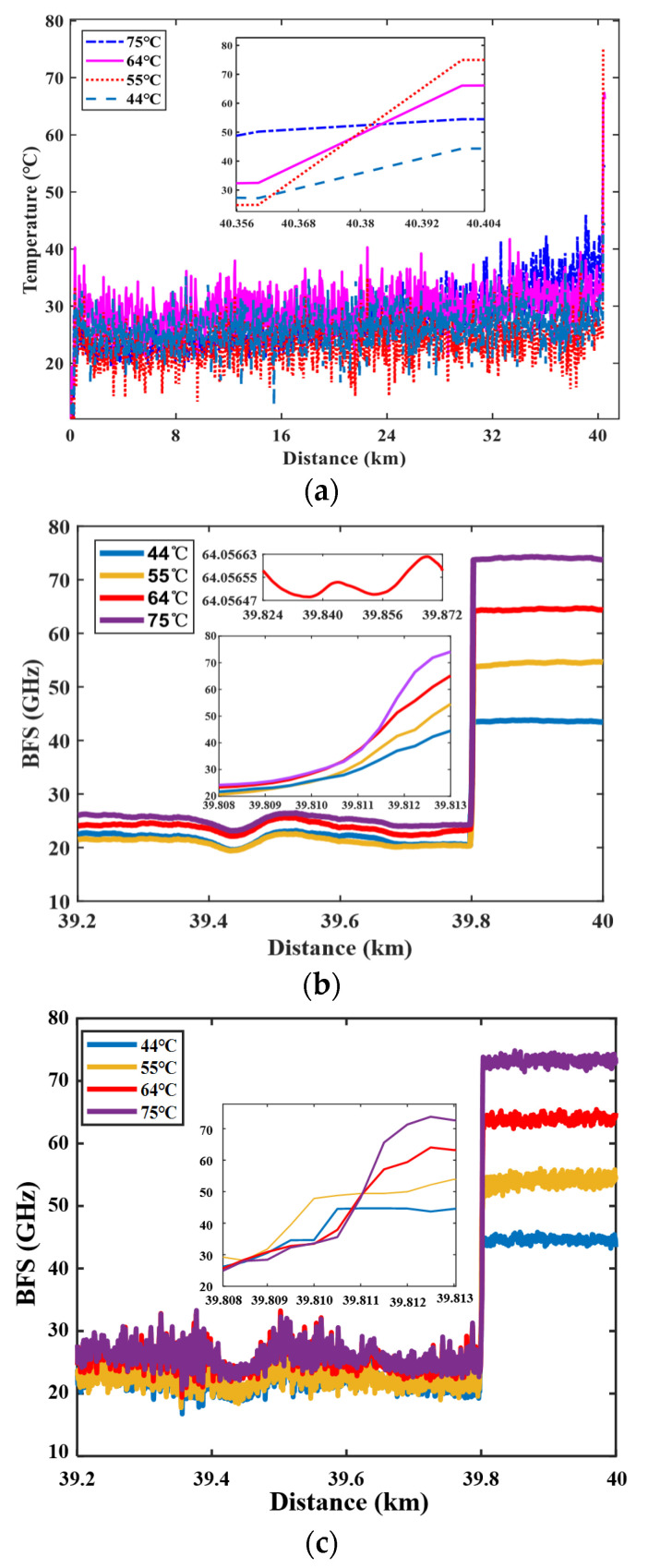
Temperature extracted from raw data, from the FNN with denoising data (**b**) and by the LCF with raw data (**c**).

When the normalized raw BGS image with a scanning frequency interval of 1 MHz is input to the FNN module, temperature information can be achieved in less than 17 s, as shown in Figure 9a. The results indicate that the extracted temperature fluctuates within a range of ±10 °C around room temperature, and the jump in the temperature transition region is clearly observed. When the input data of the FNN module result from using the QWT denoising technique, the temperature distribution can be attained in less than 17 s, and is shown in Figure 9b. The results indicate that the extracted temperature fluctuates within ±0.11 °C of the actual temperatures, 44, 55, 64 and 75 °C, which is in good agreement with the actual temperature exerted in the last 200 m fiber under test. The spatial resolution is determined as 3 m, which is consistent with the time domain width of a 15 ns pump pulse. For convenience in comparison, the temperature distribution extracted from the same data set by LCF is shown in Figure 9c. The results show that the measured temperature error maintains within ±2.5 °C for the later 200 m FUT. Notably, the initial parameters of peak point frequency and FWHM for the LCF are chosen by the BGS data at the front of the FUT. The reason for the ±2.5 °C error is that the matching parameters for LCF cannot be adjusted dynamically. Moreover, the time consumed by the QWT algorithm’s processes of denoising, normalization, linear interpolation, and temperature extraction from the FNN is less than 20 s, while for the LCF, it takes 11 m to obtain poor results. In the case of the scanning interval, no more than 6 MHz after the QWT’s denoising, normalization and linear interpolation, the BGS is input to the FNN module. The experiment data are collected at 2, 4, and 6 MHz intervals and a 200 MHz sampling rate. The room temperature is about 25 °C, and in the heat section of the FUT is 55 °C. In the transition zone, all the temperature data from the FNN fit those from the raw data completely. In addition, when the scanning interval is less than 6 MHz, the temperature error is about ±0.15 °C, which not only meets the requirements of many large infrastructures for measurement accuracy, but also can limit the time of information extraction to within 20 s.

## 4. Conclusions

In this paper, we have established a BOTDA distributed sensor system with a 40 km long sensing optical fiber to solve the data congestion problem caused by large-scale infrastructure in the process of uploading a large amount of data; it may be used for structural health monitoring of large-scale infrastructure.

Within the system, we designed the QWT algorithm to filter out the noise from the original BGS image, so as to carry out the processing and collection of the original data. After the QWT denoising algorithm is adopted, the SNR, frequency uncertainty, adaptability to scanning intervals and running time are significantly improved. For similar denoising algorithms, the QWT algorithm has comprehensive advantages in both accuracy and processing time. Then, we transferred the processed BGS data into FNN for training, in order to extract the physical information from the BGS data. The output results of the FNN show that when the frequency interval is less than 4 MHz, the temperature accuracy can reach ±0.11 °C; when the frequency interval reaches 6 MHz, the temperature accuracy is about ±0.15 °C. It takes about 17 s for the FNN module to extract the temperature distribution of the FUT. Compared with similar algorithms for extracting physical information, the artificial neural network we designed has comprehensive advantages in accuracy and processing time.

With the optimization of the QWT denoising algorithm and improvements in ANN technology, we expect that QWT image denoising technology and FNN-based information extraction methods will become useful tools for distributed structural health monitoring of large-scale infrastructure.

## Figures and Tables

**Table 1:** Comparison of running time of denoising algorithms.

Algorithm	LWT	NLM	QWT
Running Time	4 s	47 min	32 s

## Data Availability

The data presented in the present paper are available from the corresponding author upon request.
